# Systemic effects of BMP2 treatment of fractures on non-injured skeletal sites during spaceflight

**DOI:** 10.3389/fendo.2022.910901

**Published:** 2022-08-15

**Authors:** Ariane Zamarioli, Gremah Adam, Kevin A. Maupin, Paul J. Childress, Alexander Brinker, Joao P. B. Ximenez, Nabarun Chakraborty, Aarti Gautam, Rasha Hammamieh, Melissa A. Kacena

**Affiliations:** ^1^ Department of Orthopaedic Surgery, Indiana University School of Medicine, Indianapolis, IN, United States; ^2^ Department of Orthopaedics and Anaesthesiology, Ribeirão Preto Medical School, São Paulo, Brazil; ^3^ Laboratory of Molecular Biology, Blood Center of Ribeirão Preto, Medical School, São Paulo, Brazil; ^4^ Medical Readiness Systems Biology, Center for Military Psychiatry and Neuroscience, Walter Reed Army Institute of Research, Silver Spring, MD, United States; ^5^ Richard L. Roudebush VA Medical Center, Indianapolis, IN, United States

**Keywords:** fracture repair, spaceflight, microgravity, bone mass, skeleton, bone morphogenetic protein, bone healing

## Abstract

Unloading associated with spaceflight results in bone loss and increased fracture risk. Bone morphogenetic protein 2 (BMP2) is known to enhance bone formation, in part, through molecular pathways associated with mechanical loading; however, the effects of BMP2 during spaceflight remain unclear. Here, we investigated the systemic effects of BMP2 on mice sustaining a femoral fracture followed by housing in spaceflight (International Space Station or ISS) or on Earth. We hypothesized that in spaceflight, the systemic effects of BMP2 on weight-bearing bones would be blunted compared to that observed on Earth. Nine-week-old male mice were divided into four groups: 1) Saline+Earth; 2) BMP+Earth; 3) Saline+ISS; and 4) BMP+ISS (n = 10 mice/group, but only n = 5 mice/group were reserved for micro-computed tomography analyses). All mice underwent femoral defect surgery and were followed for approximately 4 weeks. We found a significant reduction in trabecular separation within the lumbar vertebrae after administering BMP2 at the fracture site of mice housed on Earth. In contrast, BMP2 treatment led to a significant increase in trabecular separation concomitant with a reduction in trabecular number within spaceflown tibiae. Although these and other lines of evidence support our hypothesis, the small sample size associated with rodent spaceflight studies limits interpretations. That said, it appears that a locally applied single dose of BMP2 at the femoral fracture site can have a systemic impact on distant bones, affecting bone quantity in several skeletal sites. Moreover, our results suggest that BMP2 treatment works through a pathway involving mechanical loading in which the best outcomes during its treatment on Earth occurred in the weight-bearing bones and in spaceflight occurred in bones subjected to higher muscle contraction.

## Introduction

The effects of spaceflight on astronaut bone mass have been well documented (1). On average, while in space, astronauts lose 1%–2% of their bone mineral density (BMD) every month (2, 3). Such a change was observed in multiple bones throughout the body, including the pelvis, spine, and vertebrae. Due to weightlessness, there is an absence of mechanical loading that would be present on Earth; thus, the skeleton is no longer under significant stress to support the body’s weight as it would be on Earth (4–6). This leads to the significant bone loss observed in spaceflight (7).

Bone morphogenetic protein 2 (BMP2) has been implicated in the development of bone. BMP2 is known to enhance bone formation through its involvement in molecular pathways associated with mechanical loading (8). Indeed, a direct interaction of BMP signaling and mechanically induced integrin signaling has been reported (9). Furthermore, others have reported a significant upregulation of alkaline phosphatase and Runx2 expression in osteoblast-like MC3T3-E1 cells by the combination of cyclic mechanical loading and BMP2 administration (10). Additionally, BMP2 contributes to phosphorylation within the Smad-dependent pathways (11). As an example, a recent investigation demonstrated that mechanical loading downregulates carbonic anhydrase IX (Car9) mRNA expression through the activation of the BMP-Smad pathway, which alkalizes the local milieu and increases alkaline phosphatase activity, thereby inducing bone formation (12). Although the exact mechanisms underlying the crosstalk between mechanical loading and BMP2 induction have not been fully dissected, previous studies have demonstrated that cyclic stretch enhanced the BMP2-induced osteoblastic differentiation through the inhibition of the Hes-related family bHLH transcription factor with YRPW motif 1 (Hey1), a potent negative regulator of osteogenesis (10). Furthermore, mechanical load has been shown to improve BMP2-induced stimulation in rat bone repair models (13). Because BMP2 is US Food and Drug Administration (FDA) approved for bone-healing indications (14), it is possible that BMP2 may be considered as a drug therapy to treat difficult fractures in space. This is especially true considering the longer-term goals to colonize the moon and/or Mars. Therefore, it is important to examine the utility of using BMP2 in spaceflight for fracture healing (fracture findings are reported in a separate article) and to also examine whether BMP2 treatment locally (at the site of the fracture) impacts distant skeletal sites differentially in spaceflight as compared to on Earth. The latter is the aim of this study. Indeed, although BMP2 is typically thought of as an important osteogenic approach to induce bone regeneration, its downstream signaling seems to depend on mechanical loading, as no significant effects were detected in hindlimb-suspended rats (15). As the osteogenic effects of BMP2 at promoting bone formation and healing are enhanced with mechanical loading by modulating cell differentiation (13), our primary hypothesis was that in spaceflight, the systemic effects of BMP2 on weight-bearing bones would be blunted compared to that observed on Earth. To test this hypothesis, we used micro-computed tomography (µCT) to analyze the bone phenotype of the following non-weight-bearing bones, calvarium, mandible, incisor, ribs, and sternum, and the following weight-bearing bones, vertebrae, humerus, and tibia.

## Materials and methods

### Animals

All animal studies were completed following the NIH Guide for the Care and Use of Laboratory Animals and were approved by the NASA Animal Care and Use Committees (#FLT-15-101/NAS-15-105).

Seven-week-old C57BL/6J male mice were shipped to Kennedy Space Center (KSC) from Jackson Laboratories (Bar Harbor, ME, USA). Fifteen mice were initially placed into each N40 mouse cage (Ancare, Bellmore, NY, USA). The mice were allo-reared, meaning they were cage-mated and kept in the same groups since weaning. The N40 cages contained a raised flooring of wire (three openings per inch). Cages contained water bottles with modified lixits (as used in NASA’s spaceflight hardware), and mice were fed NASA nutrient-upgraded rodent food bar (NuRFB). Ear-punching was used to identify mice, and body weights were recorded 2×/week to determine whether mice were acclimating to the NuRFB and water lixit system. Three mice out of 240 total mice prepared for spaceflight (in case of launch delays) were excluded from the study based on ≥10% loss of body weight. In terms of environmental conditions, the mice were kept at 24°C–25°C and on a 12-h light-and-dark cycle.

### Surgery

After 2 weeks of acclimation (mice were then 9 weeks old), cages of mice were randomized into four groups: 1) Segmental bone defect (SBD) surgery treated with saline and housed on Earth (Saline+Earth); 2) SBD surgery treated with BMP2 and housed on Earth (BMP2+Earth); 3) SBD surgery treated with saline and housed on the International Space Station or ISS (Saline+ISS); and 4) SBD surgery treated with BMP2 and housed on the ISS (BMP2+ISS). Our surgical procedure has been previously described (1, 16–19). Briefly, after being placed under ketamine-xylazine anesthesia (125–20 mg/kg), the right leg of each mouse was shaved and sterilized *via* alternating ethanol and betadine scrubs (three each). Following sterilization, a lateral 1-cm incision was made through the skin over the midshaft of the femur. A 27-gauge needle was placed between the medial and lateral femoral condyles within the intramedullary canal. After insertion, the needle was partially removed from its position. This allowed for an SBD of 2 mm to be introduced through the use of a metal Dremel saw blade (Dremel Inc., Racine, WI, USA). This 2-mm bone defect was removed from the diaphysis of the right femur. Next, a propylene fumarate/tricalcium phosphate scaffold was placed into the defect to maintain the defect size (20). The 27-gauge needle was advanced through the scaffold and the greater trochanter of the femur. At its exit site through the femur, the needle tip was bent backward. Then, a collagen sponge (RCM6 Resorbable Collagen Membrane, ACE, Brockton, MA, USA) soaked with 10 µl of saline or BMP2 (5 µg, Medtronic, Fridley, MN, USA) was placed around the scaffold. The dose of BMP2 used was empirically shown to yield significant bone healing in pilot studies (data not shown), but the range of doses tested was scaled down based on defect size, as recommended by Medtronic (personal communication MK), from those used in previous rat studies delivered by the scaffold used here (20). The collagen sponge was then sutured into place using 3–0 vicryl suture (Ethicon, Somerville, NJ, USA). The quadriceps muscle was closed by suture, and the skin incision was closed by wound clips (7 mm; Braintree Scientific, Braintree, MA, USA). The mice were watched until they recovered from the anesthetic, and all mice were provided with buprenorphine (0.05 mg/kg) as an analgesic. Once mice recovered, they were returned to their original N40 cages, which following surgery were outfitted with resting boards (Bio-Serve, K3392 Rest Stops).

### Experimental timeline

**Figure 1:**
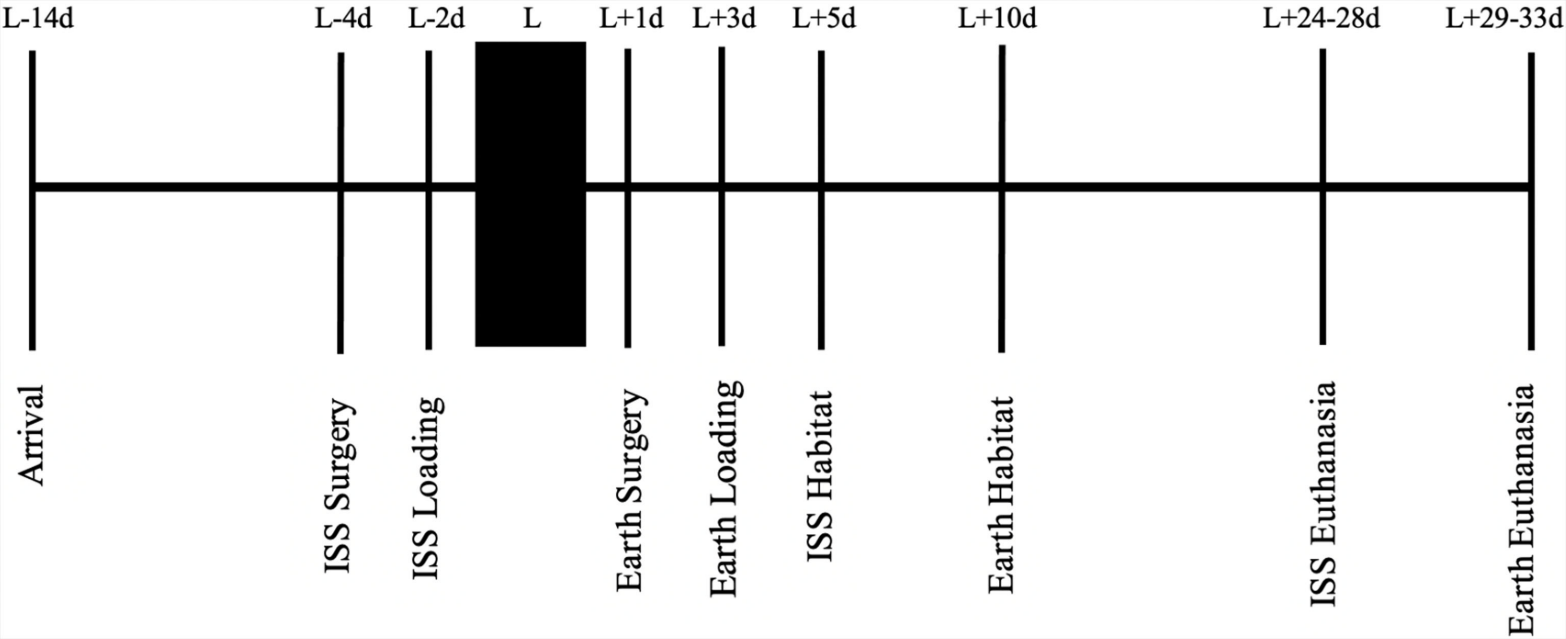
A stepwise representation of the experimental timeline, starting with mouse acclimation, to spaceflight launch, and ending with euthanasia. L, launch; d, day; ISS, International Space Station.

**Figure 1** summarizes the experimental timeline. After acclimation and surgery as described above, mice recovered from surgery for 2 days prior to being placed in NASA Transporters (spaceflight hardware used to house mice while they were on the SpaceX Dragon capsule or remained on Earth in identical hardware). Importantly, 15 mice/cage began in the study, but only the 10 “healthiest” mice continued in the study and were placed into the NASA Transporters. The general health of the mice was subjectively determined by MK and PC in consultation with NASA veterinarians and included assessment of their activity, gait, posture, and coat condition. The mice remained in the NASA Transporters for approximately 2 days prior to launch on February 19, 2017. Five days after launch, the mice were moved from NASA Transporters (10 mice/Transporter side, there are two sides to a Transporter) to NASA Habitats (five mice/Habitat side, there are two sides to a Habitat). Therefore, there are double the number of habitats on the ISS than the number of Transporters on the SpaceX Dragon.

Mice were euthanized 24–28 days after launch, when the mice were approximately 13 weeks of age. Unfortunately, the astronauts could only euthanize and process tissues from eight mice/day, requiring 5 days to euthanize all 40 mice flown for the Rodent Research 4 mission. Of note, the data presented here are part of a larger experiment, and not all specimens were available to the research team due to the multi-institutional agreements. For example, although 10 mice/group were launched into space or remained on Earth, 5 mice/group were reserved for OMICS studies and 5 mice/group were reserved for the bone analyses described here. Importantly, ground controls were asynchronous (5 days) to assist with replicating spaceflight environmental conditions that can be controlled (cage temperature, food and water changes, timing for euthanasia, etc). For example, the astronauts spent more time processing specimens in spaceflight than would be required to complete the same task on Earth. Therefore, by conducting ground controls 5 days after spaceflight studies, the researchers were able to mimic astronaut activities (e.g., replicate the duration it took to dissect specimens and store them at appropriate temperatures). Finally, to understand the BMP2 action, which is the focus of this article and to maximize the usage of unique spaceflight specimens, the data from the saline group are common to previously published studies by our group (1, 19, 21).

Of note, one mouse belonging to the Saline+ISS group was euthanized 12 days after launch based on veterinarian consultation due to the limited activity of this mouse compared to its cohort. Five mice belonging to the BMP2+ISS group were either discovered to be dead (1–7 days after launch) or were euthanized based on veterinarian consultation determining them to be grossly moribund/distressed (3–7 days after launch, 1–8 days after launch). The details surrounding the investigation into the cause of these deaths/morbid nature are detailed elsewhere (22). In brief, no conclusive cause of death was identified for the BMP2+ISS group. However, in an attempt to assist with this investigation, three healthy BMP2+Earth mice were euthanized early as controls (9 days after launch). The data presented in this article are only from mice that remained in the study until the planned euthanasia (24–28 days after launch) and for which the surgical leg was removed from the mice at the time of euthanasia (maximum of five mice/group except for BMP2+Earth group).

### Sample collection

Approximately 4 weeks post-launch, the mice were euthanized by injection of ketamine-xylazine (150–45 mg/kg), followed by a cardiac puncture to collect whole blood, and cervical dislocation. For five mice/group, the surgical hindlimb (including intact tibia analyzed here) was removed through dislocation of the hip and preserved in 10% neutral buffered formalin (NBF) and stored at 4°C. After approximately 2 weeks, the hindlimb samples were transported to Indiana University School of Medicine (IUSM) and were then washed with phosphate buffer solution (PBS). Finally, samples were placed in 70% ethanol at 4°C. The rest of the mouse carcass was enclosed in aluminum foil and frozen (at or below -80°C on the ISS). For the remaining five mice/group, after euthanasia as above, the whole mouse carcass was placed in aluminum foil and frozen. After approximately 2 weeks, the carcasses were transported on dry ice to the US Army Center for Environmental Health Research at Fort Detrick, MD, where they were stored at -80°C until processing for individual tissue isolation.

For tissue isolation, mouse carcasses were partially thawed on ice blankets for approximately 15 min before removing specific tissues. For this study, the calvaria, mandible/incisor, ribs, sternum, and vertebrae were all isolated and immediately snap frozen in liquid nitrogen and stored at -80°C prior to shipping to IUSM on dry ice. Upon arrival at IUSM, tissues were briefly thawed on ice blankets, soft tissue was removed, the bone of interest was isolated and then placed in 10% NBF for 72 h, washed with cold PBS, and finally stored in 70% ethanol at 4°C. For this study, the entire left forelimb was removed and placed in 10% NBF for 72 h, washed with cold PBS, transferred into cold 70% ethanol, and then shipped to IUSM and stored at 4°C.

### Micro-computed tomography

Imaging of tibiae was completed using a SCANCO µCT35 system (SCANCO Medical, Brüttisellen, Switzerland) at 55 kV and 12-µm voxel size with a 0.5-mm aluminum filter. For the trabecular bone analysis of the tibiae, the lower threshold was 31 and the upper threshold was 500. For total bone and midshaft analyses, the lower threshold is 240 and the upper threshold was 700. The region of interest (ROI) for trabecular analyses of tibia samples (also the tissue volume or TV) was set at 0.25 mm distal of the proximal growth plate and extended 0.5 mm proximally. Variables measured included bone volume-to-tissue volume ratio or bone volume fraction (BV/TV), trabecular separation (Tb.Sp), trabecular thickness (Tb.Th), trabecular number (Tb.N), and structure model index (SMI). Tibial cortical analyses were completed on a 1-mm ROI that was 0.25 mm proximal from the tibiofibular junction. BV and cortical thickness (Ct.Th) variables were obtained. From BV, the bone area (B.Ar) was found by dividing BV by the 1-mm height of the bone analyzed. The area equation for a cylinder, B.Ar = *(total radius^2^ - marrow radius^2^), was solved for marrow radius by substituting total radius = Ct.Th + marrow radius. Through substitution, marrow radius = [(B.Ar/π) – Ct.Th^2^)/(2*Ct.Th)]. This allowed for the calculation of the marrow area (M.Ar) = π*marrow radius^2^ and finally tissue area (T.Ar) = M.Ar + B.Ar.

A SkyScan 1172 µCT imaging system (SkyScan, Kontich, Germany) was used to image the calvarium, mandibles/incisors, ribs, sternum, vertebrae, and humeri. For the calvarium, mandibles/incisors, sternum, vertebrae, and humeri, scans were captured at 60 kV using a 5.9-μm voxel size with a 0.5-mm aluminum filter. For the ribs, scans were captured at 60 kV using a 9.8-μm voxel size. For the bones analyzed on the SkyScan, the upper threshold was 255. The lower thresholds varied as follows: for calvariae, ribs, and sternum, the lower threshold was 90; for trabecular bone within the humerus, the lower threshold was 80; for the vertebrae and cortical bone of the humerus, the lower threshold was 110; for the mandible, the lower threshold was 120. Notably, the lower threshold was set to achieve a physiologically accurate representation of each bone. All Skyscan images were reconstructed with NRecon v.1.7.3 and subsequently analyzed using Skyscan software (Dataviewer, CTAn, Kontich, Belgium).

Concerning the calvarium, the ROI was centered at the parietal eminence with a 100 pixel^3^ volume. TV, BV, BV/TV, marrow volume (MV = TV – BV), Ct.Th, Tb.h, Tb.Sp, and Tb.N were found. Calvarial thickness (Ct.Th) was calculated by averaging the width measured from three randomly selected images.

For the mandible, the ROI was a coronal slice taken midpoint through the first molar at its posterior root. The molar was removed from this ROI, and images were analyzed with the mandible and incisor together. A separate analysis was also performed on the incisor alone. Mandible variables were T.Ar, B.Ar, M.Ar (M.Ar = T.Ar-B.Ar), B.Ar/T.Ar, and CEJ-ABC. Mandible values required subtraction of corresponding incisor values [T.Ar; enamel + dentin area (E+D)Ar; pulp area or Pu.Ar = T.Ar – (E+D)Ar]. The shrink-wrap function was used to ensure accurate T.Ar measurements for the mandibles and incisors. The distance from the cementum edge on the lingual tooth surface to the apex of the alveolar bone was considered the lingual cementum–enamel to alveolar bone crest distance (CEJ-ABC).

With respect to the ribs, the 10th rib was analyzed in this study. The ROI was 0.5 mm at the midshaft of the rib. T.Ar, B.Ar, M.Ar, B.Ar/T.Ar, and Ct.Th were the cortical bone parameters collected for the rib.

Sternal and vertebral ROIs were 1-mm segments centered at the third sternal body or the L4 vertebral body, respectively. Obtained variables were TV, BV, BV/TV, BS/BV, Tb.Th, Tb.Sp, Tb.N, SMI, and Conn.D.

Finally, for the humeri, the ROI for trabecular analyses began 0.5 mm distal of the proximal growth plate and extended 0.5 mm distally. TV, BV, BV/TV, BS/BV, Tb.Th, Tb.Sp, Tb.N, SMI, and Conn.D values were obtained. With regard to cortical analyses of the humeri, the ROI was set 0.5 mm proximal of the midshaft and extended an additional 0.5 mm proximally (avoiding the deltoid tuberosity). Analysis was done for T.Ar, B.Ar, M.Ar, B.Ar/T.Ar, and Ct.Th.

As detailed above, the thresholds used for all bone analyses were selected qualitatively by an experienced operator by comparing segmented trabecular bone to original grayscale images, aiming to obtain a physiologically accurate representation. All bone morphometric parameters here described are in accordance with American Society for Bone and Mineral Research (ASBMR) nomenclature (23).

### Statistics

Data were examined for normality using the Kolmogorov–Smirnov test. Parametric data were analyzed by two-way ANOVAs to detect a significant interaction of microgravity and BMP treatment. Tukey *post-hoc* analysis was used to detect significant differences based on 1) microgravity exposure with or without treatment (e.g., Saline+Earth vs. Saline+ISS or BMP+Earth vs. BMP+ISS) or 2) BMP treatment (e.g., BMP+Earth vs. Saline+Earth or BMP+ISS vs. Saline+ISS). Statistically significant differences were noted when p < 0.05. Data were expressed as the means and standard deviations (SDs). The Benjamini–Hochberg false discovery rate was employed to control the Type I error rate from multiple comparisons. Statistical analyses were determined using RStudio 1.3 (RStudio, Inc., USA).

## Results

Because we hypothesized that in spaceflight, the systemic effects of BMP2 on weight-bearing bones would be blunted compared to that observed on Earth, we first present data on the effects of local BMP2 treatment on distant bones of mice housed on Earth and then on those housed in spaceflight. Subsequently, we show the BMP2 × microgravity interaction and examine the impacts of changing gravity [e.g., between Earth and spaceflight (ISS)] within the saline- and BMP2-treated mice.

**Tables 1** and **2** present µCT data generated from non-weight-bearing bones and weight-bearing bones, respectively. Here, the calvarium, mandible, incisor, rib, and sternum are categorized as non-weight-bearing bones (**Table 1**), while the vertebrae, humeri, and tibiae are considered weight-bearing bones (**Table 2**). The last three columns show the p-values for ANOVA significance of the BMP2 × microgravity interaction and each main effect (BMP2 or microgravity). Superscripted, like letters (e.g., ^a^, ^b^, or ^c^) on means/SD indicate when significant differences are observed based on *post-hoc* tests.

**Table 1:** μCT assessment of bone microarchitecture in non weight-bearing bones: calvarium, mandible, incisor, rib, and sternum, from mice housed both on Earth and at the International Space Station (ISS).

		EARTH		ISS		p-values		
		Saline	BMP2	Saline	BMP2	BMP2 x Microgravity	BMP2	Microgravity
CALVARIUM	TV (mm^3^)	0.058±0.003	0.055±0.006	0.053±0.005	0.052±0.004	0.81	0.50	0.29
	BV (mm^3^)	0.054±0.003	0.053±0.004	0.053±0.005	0.050±0.004	0.70	0.75	0.68
	BV/TV (%)	94.496±2.709^c^	97.522±3.488	99.370±0.116^b,c^	96.852±1.608^b^	0.07	0.17	**0.02**
	MV (mm^3^)	0.003±0.002^c^	0.001±0.002	0.0003±0.00007^b,c^	0.002±0.0009^b^	**0.002**	**0.03**	**0.002**
	Ct.Th (mm)	0.096±0.008^c^	0.110±0.009	0.117±0.100^b,c^	0.098±0.009^b^	**0.002**	0.06	**0.004**
	Tb.Th (mm)	0.075±0.006^c^	0.085±0.009	0.090±0.006^b,c^	0.076±0.007^b^	**0.002**	0.05	**0.004**
	Tb.Sp (mm)	0.004±0.002^c^	0.002±0.003	0.001±0.0001^b,c^	0.002±0.001^b^	**0.002**	**0.02**	**0.002**
	Tb.N (1/mm)	12.690±0.618^c^	11.476±0.836	11.024±0.836^b,c^	12.808±0.949^b^	**0.001**	0.05	**0.005**
MANDIBLE	T.Ar (mm^2^)	1.943±0.020	1.980±0.052	1.946±0.045	1.959±0.085	0.65	0.37	0.95
	B.Ar (mm^2^)	1.311±0.021	1.340±0.066	1.337±0.041	1.350±0.055	0.74	0.43	0.48
	M.Ar (mm^2^)	0.632±0.041	0.640±0.054	0.609±0.029	0.610±0.036	0.85	0.81	0.43
	B.Ar/T.Ar (%)	67.498±1.781	67.694±2.718	68.719±1.336	68.925±0.881	0.98	0.89	0.34
	CEJ-ABC (mm)	0.192±0.008	0.207±0.026	0.221±0.027	0.198±0.012	0.08	0.32	0.07
INCISOR	T.Ar (mm^2^)	0.467±0.005	0.465±0.012	0.481±0.015	0.481±0.012	0.89	0.84	0.09
	E+D.Ar (mm^2^)	0.377±0.034	0.355±0.020^c^	0.394±0.037	0.385±0.020^c^	0.58	0.24	0.39
	Pu.Ar (mm^2^)	0.090±0.029	0.110±0.020	0.087±0.023	0.096±0.015	0.60	0.16	0.89
	E+D.Ar/T.Ar (%)	80.709±6.539	76.421±4.173	81.742±5.355	80.036±3.243	0.53	0.18	0.74
RIB	T.Ar (mm^2^)	0.088±0.024	0.101±0.007	0.114±0.013^b^	0.089±0.008^b^	**0.03**	0.15	**0.02**
	B.Ar (mm^2^)	0.069±0.017	0.075±0.005	0.083±0.008^b^	0.067±0.007^b^	0.45	0.70	0.46
	M.Ar (mm^2^)	0.019±0.008	0.026±0.005	0.031±0.005^b^	0.022±0.002^b^	0.11	0.20	0.09
	B.Ar/T.Ar (%)	78.766±3.455^c^	73.914±3.975	72.875±2.140^c^	75.343±1.896	0.06	0.06	**0.03**
	Ct.Th (mm)	0.081±0.006	0.079±0.006	0.081±0.002	0.077±0.005	0.74	0.66	0.87
STERNUM	TV (mm^3^)	0.527±0.029	0.515±0.039	0.560±0.045	0.541±0.045	0.90	0.70	0.34
	BV (mm^3^)	0.058±0.012	0.044±0.013	0.051±0.006	0.044±0.006	0.44	**0.04**	0.37
	BV/TV (%)	10.994±1.647^a,c^	8.351±1.953^a^	9.047±0.751^c^	8.043±0.932	0.25	**0.009**	0.07
	BS/BV (%)	0.084±0.004	0.096±0.011	0.088±0.002	0.090±0.008	0.07	**0.007**	0.30
	Tb.Th (mm)	0.039±0.002	0.037±0.003	0.038±0.001	0.040±0.003	**0.03**	**0.048**	0.30
	Tb.Sp (mm)	0.211±0.017	0.212±0.029	0.214±0.022	0.243±0.019	0.15	1.0	0.84
	Tb.N (1/mm)	2.770±0.294^c^	2.268±0.517	2.380±0.239^c^	2.038±0.285	0.67	**0.03**	0.12
	SMI	1.718±0.168^a^	2.113±0.310^a^	1.774±0.119	1.973±0.176	0.28	**0.004**	0.59
	Conn.D (1/um^3^)	0.028±0.008	0.032±0.003	0.026±0.005	0.024±0.015	0.89	0.66	0.85

TV, tissue volume; BV, bone volume; BV/TV, bone volume fraction; MV, marrow volume, calculated as TV-BV; Ct.Th, average cortical thickness; Tb.Th, trabecular thickness; Tb.Sp, trabecular separation; Tb.N, trabecular number; T.Ar, tissue area; B.Ar, bone area; M.Ar, marrow area, calculated as T.Ar-B.Ar; B.Ar/T.Ar, bone area fraction; CEJ-ABC, lingual cementum–enamel to alveolar bone crest distance; E+D.Ar, enamel + dentin area; Pu.Ar, pulp area; E+D.Ar/T.Ar, enamel + dentin area fraction; BS/BV, specific bone surface; SMI, structure model index; Conn.D, connectivity density. Sample sizes for all bones are n=5 unless otherwise indicated, Calvarium (BMP+Earth, n=3, Saline+ISS, n=4), Mandible and Incisor (Saline+Earth, n=3, Saline+ISS, n=4), Rib (Saline+ISS, n=4, BMP+ISS, n=3). Significant interactions and main effects were detected by 2-way ANOVA and p-values are shown. Tukey post-hoc analyses were used to detect significant differences based on, 1) BMP2 versus Saline treatment on Earth (^a^ indicates significant differences between BMP2+Earth versus Saline+Earth, p<0.05); 2) BMP2 versus Saline treatment in spaceflight (^b^ indicates significant differences between BMP2+ISS versus Saline+ISS, p<0.05); 3) microgravity exposure with identical treatments (^c^ indicates significant differences between identified Earth and ISS groups, p<0.05) Values are expressed as mean ± S.D.

**Table 2:** μCT assessment of bone microarchitecture in weight bearing bones: fourth lumbar vertebrae (L4), humerus, and tibias, from mice housed both on Earth and at the International Space Station (ISS).

		EARTH		ISS			p-values	
		Saline	BMP2	Saline	BMP2	BMP2 x Microgravity	BMP2	Microgravity
L4	TV (mm^3^)	0.950±0.245	1.079±0.080	1.110±0.086	1.086±0.034	0.22	0.12	0.07	
	BV (mm^3^)	0.181±0.066	0.226±0.032	0.176±0.024	0.189±0.015	0.36	0.09	0.76	
	BV/TV (%)	18.516±4.072	20.904±1.721^c^	15.865±1.510	17.433±1.680^c^	0.71	0.11	0.12	
	BS/BV (%)	70.457±10.017	67.644±2.743	78.063±4.077	71.865±4.843	0.50	0.50	**0.04**	
	Tb.Th (mm)	0.048±0.006	0.052±0.004	0.045±0.004	0.049±0.005	0.93	0.17	0.26	
	Tb.Sp (mm)	0.200±0.006^a^	0.184±0.010^a,c^	0.210±0.016	0.205±0.006^c^	0.41	0.12	0.40	
	Tb.N (1/mm)	3.777±0.395	4.001±0.235^c^	3.501±0.176	3.581±0.173^c^	0.61	0.24	0.16	
	SMI	1.274±0.114	1.241±0.097	1.222±0.105	1.301±0.162	0.50	0.78	0.65	
	Conn.D (1/um^3^)	0.178±0.079 ^a,c^	0.055±0.010^a^	0.050±0.008^c^	0.047±0.005	**0.03**	**0.002**	**0.0007**	
TRABECULAR HUMERUS	TV (mm^3^)	0.659±0.052	0.717±0.006	0.661±0.052	0.716±0.060	0.91	**0.03**	0.87	
	BV (mm^3^)	0.083±0.033	0.113±0.033	0.074±0.029	0.075±0.027	0.26	0.05	0.55	
	BV/TV (%)	12.603±5.249	15.704±3.919^c^	11.312±4.635	10.434±3.296^c^	0.26	0.12	0.52	
	BS/BV (%)	68.294±9.754	64.077±5.328	66.825±10.167	64.420±5.449	0.80	0.31	0.74	
	Tb.Th (mm)	0.062±0.006	0.065±0.003	0.063±0.007	0.065±0.004	0.92	0.42	0.91	
	Tb.Sp (mm)	0.240±0.043	0.207±0.020^c^	0.274±0.059	0.300±0.005^c^	0.05	0.10	0.10	
	Tb.N (1/mm)	1.962±0.705	2.418±0.537^c^	1.739±0.589	1.590±0.467^c^	0.16	0.09	0.41	
	SMI	2.585±0.218	2.472±0.180	2.586±0.142	2.645±0.205	0.16	0.17	0.95	
	Conn.D (1/um^3^)	0.282±0.139	0.408±0.137^c^	0.181±0.087	0.186±0.083^c^	0.35	0.12	0.07	
CORTICAL HUMERUS	T.Ar (mm^2^)	0.913±0.048	0.928±0.061	0.946±0.028	0.927±0.055	0.34	0.55	0.13	
	B.Ar (mm^2^)	0.548±0.041	0.560±0.040	0.544±0.030	0.528±0.054	0.33	0.55	0.85	
	M.Ar (mm^2^)	0.365±0.028^c^	0.367±0.022^c^	0.403±0.033^c^	0.400±0.019^c^	0.78	0.82	**0.005**	
	B.Ar/T.Ar (%)	60.015±2.632	60.379±0.656^c^	57.459±2.967	56.843±2.771^c^	0.60	0.76	**0.04**	
	Ct.Th (mm)	0.181±0.013	0.184±0.008	0.176±0.012	0.170±0.016	0.35	0.64	0.36	
TRABECULAR TIBIA	TV (mm^3^)	1.339±0.083	1.341±0.220	1.461±0.101	1.436±0.180	0.90	0.91	0.26	
	BV (mm^3^)	0.241±0.043	0.278±0.129	0.277±0.136	0.174±0.072	0.16	0.74	0.85	
	BV/TV (%)	18.068±3.463	20.504±7.816	18.918±8.874	12.312±2.175	0.17	0.69	0.87	
	Tb.Th (mm)	0.047±0.005	0.056±0.013	0.055±0.014	0.048±0.005	0.12	0.21	0.31	
	Tb.Sp (mm)	0.145±0.012	0.152±0.013^c^	0.160±0.013^b^	0.201±0.028^b,c^	**0.008**	0.44	0.14	
	Tb.N (1/mm)	6.348±0.464^c^	6.093±0.400^c^	5.787±0.291^b.c^	4.931±0.590^b,c^	0.11	0.46	0.11	
	SMI	2.353±0.301	2.330±0.408	2.290±0.509	2.666±0.326	0.23	0.88	0.71	
	Conn.D (1/mm^3^)	204.232±55.828	170.996±32.176^c^	141.417±46.682	99.650±57.455^c^	0.43	0.55	0.17	
CORTICAL TIBIA	T.Ar (mm^2^)	0.989±0.041	0.989±0.127	1.064±0.070	0.997±0.070	0.42	0.92	0.21	
	B.Ar (mm^2^)	0.676±0.043	0.681±0.113	0.738±0.053	0.679±0.072	0.40	0.98	0.25	
	M.Ar (mm^2^)	0.313±0.010	0.308±0.027	0.326±0.026	0.318±0.008	0.69	0.65	0.17	
	B.Ar/T.Ar (%)	53.265±3.475	53.900±2.949	55.622±3.731	53.609±2.293	0.35	0.75	0.27	
	Ct.Th (mm)	0.246±0.014	0.250±0.028	0.258±0.013	0.244±0.021	0.42	0.89	0.43	
	Density TV (1/cm)	3.029±0.209	3.009±0.190	3.154±0.133	3.000±0.111	0.38	0.86	0.29	
	Density BV (1/cm)	5.117±0.118	5.016±0.139	5.087±0.046	5.040±0.063	0.56	0.15	0.69	

TV, tissue volume; BV, bone volume; BV/TV, bone volume fraction; BS/BV, specific bone surface; Tb.Th, trabecular thickness; Tb.Sp, trabecular separation; Tb.N, trabecular number; SMI, structure model index; Conn.D, connectivity density; T.Ar, tissue area; B.Ar, bone area; M.Ar, marrow area; B.Ar/T.Ar, bone area fraction; Ct.Th, average cortical thickness. Sample sizes for L4 are n=4 for all groups, with the exception of Saline+Earth, n=5. For humerus sample sizes are n=10, with the exception of BMP+Earth, n=6 and BMP+ISS, n=5. Sample sizes for tibias are n=5 for all groups, except for BMP+ Earth, n=6 (due to unexpected euthanasia, some samples were not reserved for OMICS analyses so an extra sample was available for some bones). Significant interactions and main effects were detected by 2-way ANOVA and p-values are shown. Tukey post-hoc analyses were used to detect significant differences based on, 1) BMP2 versus Saline treatment on Earth (^a^ indicates significant differences between BMP2+Earth versus Saline+Earth, p<0.05); 2) BMP2 versus Saline treatment in spaceflight (^b^ indicates significant differences between BMP2+ISS versus Saline+ISS, p<0.05); 3) microgravity exposure with identical treatments (^c^ indicates significant differences between identified Earth and ISS groups, p<0.05) Values are expressed as mean ± S.D.

### The effects of bone morphogenetic protein 2 treatment on mice housed on Earth

**Figure 2:**
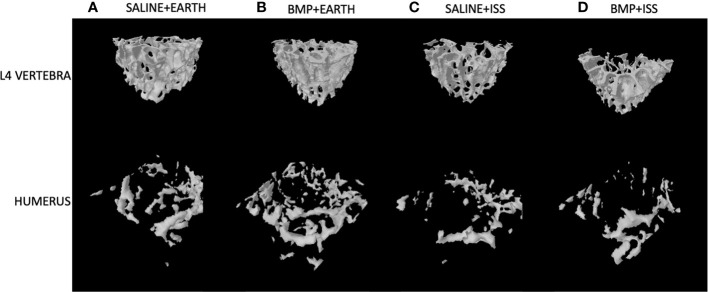
Representative 3D µCT reconstructions of the trabecular compartment of vertebral and humeral bone in the following groups: **(A)** Saline+Earth; **(B)** BMP+Earth; **(C)** Saline+ISS; and **(D)** BMP+ISS. BMP, Bone Morphogenetic Protein 2; ISS, International Space Station; L, lumbar verterbrae; uCT, micro-computed tomography.

First, we examined the impact of BMP2 vs. saline administered locally at the fracture site on other skeletal locations for mice housed on Earth (significant differences are indicated by the letter ^a^ in **Tables 1** and **2**). With regard to significant effects on non-weight-bearing bones, BMP2 treatment on Earth resulted in a significant 24% reduction in trabecular BV/TV (p = 0.05) and a 23% increase in SMI (p = 0.04) in the sternum. Related to weight-bearing bones, compared to saline treatment, BMP2 induced a significant 8% decrease in Tb.Sp (p = 0.02) within the L4 vertebra, despite a significant 69% decrease in connectivity density (Conn.D, p = 0.02). No other significant BMP2 treatment effects were observed for mice housed on Earth. **Figure 2** illustrates representative tridimensional images of trabecular bone within the L4 vertebra and humerus for all analyzed groups.

### The effects of bone morphogenetic protein 2 treatment on mice housed on the international space station

Second, we examined the impact of BMP2 vs. saline administered locally at the fracture site on other skeletal locations for mice housed in spaceflight on the ISS (significant differences are indicated by the letter ^b^ in **Tables 1** and **2**). For non-weight-bearing bones, compared to saline treatment, BMP2 treatment resulted in a significant 3% reduction in calvarium BV/TV (p = 0.02), which was associated with a 567% increase in MV (p = 0.03), a 16% reduction in both cortical and trabecular thickness (Ct.Th, p = 0.02; Tb.Th, p = 0.01, respectively), a 100% increase in Tb.Sp (p = 0.01), and a 16% increase in Tb.N (p = 0.02). For ribs, compared to saline treatment, BMP2 treatment led to a significant 22% decrease in T.Ar (p = 0.03) and a 19% decrease in B.Ar (p = 0.04), which resulted in a 29% reduction in M.Ar (p = 0.04). With respect to weight-bearing bones, compared with saline treatment, BMP2 treatment resulted in a significant 26% increase in Tb.Sp (p = 0.02), which was associated with a 15% decrease in Tb.N (p = 0.02) in the tibiae. No other significant BMP2 treatment effects were observed for mice housed on the ISS.

### The effects of bone morphogenetic protein 2 treatment and its interaction with spaceflight

We also examined the interaction of BMP2 treatment with microgravity. Significant interactions were detected for the calvarial MV (p = 0.002), Ct.Th (p = 0.002), Tb.Th (p = 0.002), Tb.Sp (p = 0.002), and Tb.N (p = 0.001); rib T.Ar (p = 0.03); sternum Tb.Th (p = 0.03); vertebral Conn.D (p = 0.03); and the tibial Tb.Sp (p = 0.008) (**Tables 1**, **2**). For the sternum, a significant main effect of BMP2 was detected for BV (p = 0.04), BV/TV (p = 0.009), BS/BV (0.007), Tb.N (p = 0.03), and SMI (p = 0.004). For the trabecular compartment of the humerus, a significant main effect of BMP2 was detected for TV (p = 0.03). For the cortical compartment of the humerus, a significant main effectof microgravity was detected for M.Ar (p = 0.005) and B.Ar/T.Ar (p = 0.04).

Next, we examined the impacts of changing gravity within the same treatment (e.g., saline or BMP2) by comparing results from mice housed on Earth vs. the ISS (significant differences are indicated by the letter ^c^ in **Tables 1** and **2**). With saline treatment, the lack of gravity led to a 5% increase in BV/TV (p = 0.009) and a 20% increase in Tb.Th (p = 0.006) in the calvarium, which was associated with a 90% decrease in MV (p = 0.01) and an 87% decrease in Tb.Sp (p = 0.008). Interestingly, there was also a 13% reduction in Tb.N (p = 0.01), which may indicate that enough bone was formed between calvarial trabeculae to fuse them likely due to enhanced mechanical forces caused by fluid shift. On the other hand, saline-treated mice housed on the ISS exhibited a significant 7% reduction in B.Ar/T.Ar (p = 0.02) at the rib, a significant 18% decrease in BV/TV (p = 0.05) with an associated 14% decrease in Tb.N (p = 0.05) within the sternum, a significant 72% decrease in Conn.D (p = 0.02) within the vertebra, a significant 10% increase in M.Ar (p = 0.02) within the cortical compartment of the humerus, and a 9% decrease in Tb.N (p = 0.05) within the tibia likely due to the weightless condition and lack of mechanotransduction signaling.

**Figure 3:**
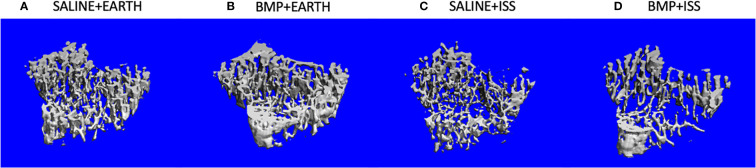
Representative 3D µCT reconstructions of the trabecular compartment of tibiae in the following groups: **(A)** Saline+Earth; **(B)** BMP+Earth; **(C)** Saline+ISS; and **(D)** BMP+ISS. BMP, Bone Morphogenetic Protein 2; ISS, International Space Station; uCT, micro-computed tomography

With BMP2 treatment, exposure to microgravity led to a significant 8% increase in E+D.Ar (p = 0.049) in the incisor; a significant 17% decrease in BV/TV (p = 0.03), an 11% increase in Tb.Sp (p = 0.01), and a 10% reduction in Tb.N (p = 0.03) in the L4 vertebra; a significant 45% increase in Tb.Sp (p = 0.002), a significant 34% decrease in Tb.N (p = 0.02), and a significant 54% decrease in Conn.D (p = 0.01) in the trabecular compartment of the humerus; a significant 9% increase in M.Ar (p = 0.03) and a 6% decrease in B.Ar/T.Ar (p = 0.01) in the cortical compartment of the humerus; and a significant 32% increase in Tb.Sp (p = 0.007), a 19% decrease in Tb.N (p = 0.005), and a 42% decrease in Conn.D (p = 0.04) in the trabecular compartment of the tibia. **Figure 3** illustrates representative tridimensional images of trabecular bone within the tibia for all analyzed groups.

## Discussion

Bone healing during spaceflight may represent a major impact in medical science in the near future due to longer missions and space colonization. We have previously demonstrated that spaceflight leads to disrupted formation-related microarchitecture parameters in several skeletal sites (1). Here, our treatment control groups (Saline) evidenced an increase in M.Ar and a decrease in B.Ar fraction within the rib, a decreased BV/TV and Tb.N within the sternum, a decreased Conn.D within the vertebra, an increased M.Ar at the cortical humerus, and a reduction in Tb.N within the tibia when mice were housed in spaceflight as compared to on Earth.

Due to the lack of gravity and physiological and molecular changes in bone cells during their differentiation, the use of osteogenic factors may represent an effective approach to enabling bone regeneration during space missions (23). Because BMP2 has been approved for several bone regeneration indications by the FDA and the European Medicines Agency (24, 25), it may be considered a likely therapy for spaceflight bone regeneration. Indeed, a recent study demonstrated the correlation between simulated microgravity with an important reduction in osteogenic in BMP2-induced osteogenetic differentiation (25). BMPs are multifunctional growth factors belonging to the transforming growth factor beta (TGF-β) superfamily and are responsible for several functions during skeletal development, bone formation, and bone repair (26). Proper bone formation requires the differentiation of osteoblasts from mesenchymal stem cell precursors, a process mediated in part by BMP signaling (26).

Under simulated microgravity, Qin et al. (27) recently evidenced a marked increase in the expression of a certain miRNA, miR-494, which is related to osteogenesis inhibition by direct suppression of two pivotal factors in BMP signaling: BMPR2 and RUNX2. Similar outcomes were also obtained by true microgravity in which the expression of BMP2 was decreased during spaceflight, leading to inhibited osteogenic differentiation (28). This highlights the action of BMP2 through mechanical loading pathways (11).

The crosstalk between mechanotransduction and BMP signaling was shown by Kopf et al. (11). The authors demonstrated that mechanical signals are integrated into the BMP signaling pathway, thus enhancing immediate early steps within the Smad pathway. The authors also suggested that other growth factor pathways, such as Wnt or insulin-like growth factor signaling, which share many downstream partners and target genes with the BMP pathway, are also influenced by mechanical loading and might be involved in BMP pathway regulation (11). Likewise, a recent study suggested that the administration of BMP2 concomitant with mechanical loading may improve bone healing by modulating early angiogenic and cytokine signaling, promoting endochondral ossification (29). Taken together, these studies demonstrate the importance of mechanical loading in BMP2-mediated bone regeneration.

Thus, although BMP2 may be a promising drug to be added to a “medical kit” when colonizing the Moon and Mars, it is also important to understand how the local administration of BMP2 could systemically impact other bones. Here, we investigated the systemic effects of a local administration of BMP2 at the femoral fracture gap on distant bones in 9-week-old C57BL/6J male mice submitted to a 4-week-period of microgravity.

As shown in **Table 2**, in agreement with our hypothesis that, in spaceflight, the systemic effects of BMP2 on weight-bearing bones would be blunted compared to that observed on Earth, we found a significant decrease in Tb.Sp within the vertebrae of mice treated with BMP2 vs. saline and housed on Earth. In contrast, when housed on the ISS, BMP2 treatment did not alter Tb.Sp within the L4 vertebra of mice. Although statistical significance was not reached with our small sample size, a similar osteogenic promoting trend was observed within the trabecular bone compartment of the humerus and tibia of mice treated with BMP2 compared to those treated with saline and housed on Earth. Specifically, a trending 25% increase in trabecular BV/TV was observed in the humerus of mice treated with BMP2 compared to saline and housed on Earth. However, when housed on the ISS, an 8% reduction in trabecular BV/TV was observed in the humerus of mice treated with BMP2 as compared to those treated with saline. Similar results were seen for trabecular BV of the humerus. Likewise, when compared to saline treatment, BMP2 treatment appeared to increase BV, BV/TV, and Tb.Th within the trabecular compartment of the tibia when mice were housed on Earth, but reductions were observed in these same parameters for these groups housed on the ISS.

On the other hand, as shown in **Table 1**, but also in agreement with our hypothesis, BMP2 treatment did not lead to any positive osteogenic effects in non-weight-bearing bone when mice were housed on Earth. Indeed, the only significant difference observed was with the sternum where a significant reduction in BV/TV was observed concomitant with a higher SMI, which indicates that the bone structures are moving from plate toward rod-like structures, in which higher values have been related to increased bone loss (30). In the absence of gravity, compared to treatment with saline, BMP2 treatment resulted in negative changes within several non-weight-bearing bones. For example, for mice housed in microgravity, within the calvarium, BMP2 treatment resulted in decreased BV/TV, which was associated with an increase in MV, as well as reductions in both Ct.Th and Tb.Th, and an increase in Tb.N. Indeed, as shown in **Table 1**, the magnitude of the statistical differences detected in the calvarium between BMP+ISS and Saline+ISS specimens is striking. While not formally tested here, these dramatic results may be due to the well-documented cephalic shift in fluids observed in spaceflight (31) and may suggest an impact of osteocyte canalicular fluid flow networks as part of the mechanism of action. A future study will be required to follow up on these interesting observations.

Likewise, for mice housed on the ISS, compared to saline treatment, BMP2 treatment resulted in a decrease in B.Ar within the ribs. While we recognize that not all bones followed these trends, it is nonetheless important to consider that even with a small sample size, significant differences are being observed in bones distant to the local treatment of a femoral defect with BMP2 and that several differences are seen based on whether the mice were housed on Earth or in spaceflight, implicating gravity-dependent mechanisms.

When comparing the systemic effects of BMP2 on Earth vs. space specimens, BMP2-treated mice in space trended toward higher tissue and enamel plus dentin area at the incisor than mice treated on Earth. We believe that these results may be due to a cephalic shift of fluid caused by spaceflight, leading to high intracranial pressure (31). Angiogenesis precedes osteogenesis, and this empirical observation has led to the suggestion that angiogenesis plays an active role in the process of osteogenesis, which is enhanced by BMP2 (32). The cephalic shift of fluid may also explain the higher bone volume fraction and thickness, concomitant with lower Tb.Sp detected in the saline group subjected to spaceflight when compared to the saline group housed on Earth. This is consistent with our previous study indicating an increase in calvarial BV (19).

A recent investigation correlated bone changes in mice following a 1-month spaceflight mission to those due to the aging process, including osteocyte cell death, lacunae mineralization, and fatty marrow. The authors also showed that changes persisted after returning to Earth for 8 days, which represented 27% of the duration of the spaceflight. This finding may suggest that spaceflight results in a chronic impairment at the level of tissue maintenance and repair (33); this further highlights the impact of these changes on increasing fracture risk. In this study, we demonstrated an important osteogenic effect of BMP2 concomitant with mechanical loading stimulus, which appears to happen not only in the presence of gravity but also during muscle contraction during spaceflight. Therefore, this highlights an important role of the bone–muscle crosstalk in improving bone formation through BMP2. Previous authors have also demonstrated the ability of some BMPs (mainly BMP2) to induce satellite cell activation and myofiber formation (34, 35). In fact, Scimeca et al. (36) reported the role of BMPs and myostatin pathways in physiopathogenesis of human sarcopenia, which may also represent a possible therapeutic option for the musculoskeletal changes due to microgravity exposure. Taken together, while BMP2 treatment remains a good option for bone regeneration on Earth, when administered locally, one time, at concentrations suitable for bone repair, as completed in these studies, it has limited osteogenic effects on distant bones, with a few notable exceptions. This suggests that other therapies will be required to stimulate anabolic bone formation in spaceflight.

Finally, it is important to acknowledge that spaceflight is a unique, yet challenging, laboratory environment for science investigations using mice. Additionally, although the hindlimb suspension model has been used to mimic many aspects of spaceflight to circumvent these issues, some differences have been noted (37). Thus, we felt that it was important to share with the greater scientific community our many results presented in **Tables 1** and **2**, even though we are underpowered to detect significant differences for several parameters. As expected, there are several barriers to conducting research in space, including limited research capabilities in launch vehicles and housing rodents in space (maximum of 40 mice are able to be housed in NASA equipment), significant limitations in what type of research activities can occur, the time it takes for research activities to occur, limitations for adjusting studies based on findings, reduced ability to readily repeat studies, and of course the exuberant costs. Therefore, a major limitation of this study is related to its sample size. Although statistical tests for sample size calculation are important and necessary to achieve statistical power, some experiments, like spaceflight investigations, cannot sustain larger sample sizes; however, findings remain important.

## Conclusions

In conclusion, our findings indicate that a single and local administration of BMP2 at the femoral fracture gap can have a systemic impact on distant bones, resulting in improved bone quantity in several skeletal sites. Moreover, our results suggest that BMP2 treatment appears to work through a pathway involving mechanical loading, in which the best outcomes during its treatment on Earth occurred in the weight-bearing bones likely owing to gravity, and in spaceflight, the best results occurred in bones subjected to higher muscle contraction.

## Data availability statement

The raw data supporting the conclusions of this article will be made available by the authors, without undue reservation.

## Ethics statement

The animal study was reviewed and approved by NASA Animal Care and Use Committees (#FLT-15-101/NAS-15-105).

## Author contributions

AZ and GA drafted the initial manuscript and together with JX created all tables/figures. AZ, GA, KM, PC, and AB completed µCT scanning and analyses. Statistical analyses were completed by JX. PC, NC, AG, RH, and MK performed the surgeries and together with KM they completed the tissue dissections. The idea conception, experimental design, identification of funding sources, and majority of logistics were completed by NC, AG, RH, and MK. All authors contributed to the article and approved the submitted version.

## Funding

This study is made possible *via* support from NIH Training Grants T32 DK007519 (P.J.C.) and T32 AR065971 (K.A.M.), the Orthopaedic Trauma Association (M.A.K.), the Ralph W. and Grace M. Showalter Research Trust Fund (M.A.K.), GA-2015-217 from the Center for the Advancement of Sciences in Space (M.A.K.), and NIH/NIAMS R01 AR060863 (M.A.K.). This material is also the result of work supported with resources and the use of facilities at the Richard L. Roudebush VA Medical Center, Indianapolis, IN: VA Merit #BX003751 (M.A.K.). In addition, support was provided by the São Paulo Research Foundation (FAPESP, #25606-4 to A.Z.). Support was also received from the U.S. Army Medical Research and Material Command (N.C., A.G., R.H.). 

## Acknowledgments

We extend our gratitude to trainees, investigators, and staff from IUSM and the U.S. Army who assisted in this study. We thank the Department of Defense Space Test Program, in particular Perry Ballard, Carolynn Conley, and James McLeroy. And we thank each member of the NASA Rodent Research 4 group, all NASA supporting personnel, and the astronauts of the ISS (Increment 50). The contents of this study are the sole responsibility of its authors and are not necessarily reflective of the official views of the aforementioned organizations. Material has been reviewed by the Walter Reed Army Institute of Research. There is no objection to its presentation and/or publication. The opinions or assertions contained herein are the private views of the author, and are not to be construed as official, or as reflecting true views of the Department of the Army or the Department of Defense. Research was conducted under an approved animal use protocol in an AAALAC International-accredited facility in compliance with the Animal Welfare Act and all other federal statutes and regulations relating to animals and experiments involving animals, and adheres to principles stated in the Guide for Care and Use of Laboratory Animals, NRC Publication, 2011 editions (38).

## Conflict of interest

The authors declare that the research was conducted in the absence of any commercial or financial relationships that could be construed as a potential conflict of interest.

## Publisher’s note

All claims expressed in this article are solely those of the authors and do not necessarily represent those of their affiliated organizations, or those of the publisher, the editors and the reviewers. Any product that may be evaluated in this article, or claim that may be made by its manufacturer, is not guaranteed or endorsed by the publisher.

## Abbreviations

CEJ-ABC, alveolar bone crest distance; B.Ar, bone area; BMP2, bone morphogenetic protein 2; BV, bone volume; BV/TV, bone volume fraction; Conn.D, connectivity density; Ct.Th, cortical thickness; Cs.Th, cross-sectional thickness; (E+D)Ar, enamel + dentin area; µCT, micro-computed tomography; M.Ar, marrow area; MV, marrow volume; NuRFB, NASA nutrient-upgraded rodent food bar; NBF, neutral buffered formalin; PBS, phosphate buffer solution; Pu.Ar, pulp area; ROI, region of interest; SBD, segmental bone defect; BS/BV, specific bone surface; SMI, structure model index; T.Ar, tissue area; TV, total volume; Tb.Sp, trabecular separation; Tb.Th, trabecular thickness; Tb.N, trabecular number.

## References

[1] Dadwal UC Maupin KA Zamarioli A Tucker A Harris JS Fischer JP. The effects of spaceflight and fracture healing on distant skeletal sites. Sci Rep (2019) 9(1):11419. doi: 10.1038/s41598-019-47695-3 31388031 PMC6684622

[2] Collet P Uebelhart D Vico L Moro L Hartmann D Roth M. Effects of 1- and 6-month spaceflight on bone mass and biochemistry in two humans. Bone (1997) 20(6):547–51. doi: 10.1016/s8756-3282(97)00052-5 9177869

[3] Vico L Hargens A. Skeletal changes during and after spaceflight. Nat Rev Rheumatol (2018) 14(4):229–45. doi: 10.1038/nrrheum.2018.37 29559713

[4] Goodship AE Cunningham JL Oganov V Darling J Miles AW Owen GW. Bone loss during long term space flight is prevented by the application of a short term impulsive mechanical stimulus. Acta Astronaut (1998) 43(3-6):65–75. doi: 10.1016/s0094-5765(98)00144-1 11541937

[5] Amin S. Mechanical factors and bone health: Effects of weightlessness and neurologic injury. Curr Rheumatol Rep (2010) 12(3):170–6. doi: 10.1007/s11926-010-0096-z PMC4533914 20425519

[6] Demontis GC Germani MM Caiani EG Barravecchia I Passino C Angeloni D. Human pathophysiological adaptations to the space environment. Front Physiol (2017) 8:547. doi: 10.3389/fphys.2017.00547 28824446 PMC5539130

[7] Williams D Kuipers A Mukai C Thirsk R. Acclimation during space flight: Effects on human physiology. Cmaj (2009) 180(13):1317–23. doi: 10.1503/cmaj.090628 PMC2696527 19509005

[8] Schwarz C Ott CE Wulsten D Brauer E Schreivogel S Petersen A. The interaction of Bmp2-induced defect healing in rat and fixator stiffness modulates matrix alignment and contraction. JBMR Plus (2018) 2(3):174–86. doi: 10.1002/jbm4.10031 PMC6124159 30283901

[9] Zong ming W Jian yu L Rui xin L Hao L Yong G Lu L. Bone formation in rabbit cancellous bone explant culture model is enhanced by mechanical load. BioMed Eng Online (2013) 12:35. doi: 10.1186/1475-925x-12-35 23597232 PMC3651399

[10] Zeng Z Yin X Zhang X Jing D Feng X. Cyclic stretch enhances bone morphogenetic protein-2-Induced osteoblastic differentiation through the inhibition of Hey1. Int J Mol Med (2015) 36(5):1273–81. doi: 10.3892/ijmm.2015.2354 PMC4601743 26647760

[11] Kopf J Petersen A Duda GN Knaus P. Bmp2 and mechanical loading cooperatively regulate immediate early signalling events in the bmp pathway. BMC Biol (2012) 10:37. doi: 10.1186/1741-7007-10-37 22540193 PMC3361481

[12] Miyamoto S Yoshikawa H Nakata K. Axial mechanical loading to ex vivo mouse long bone regulates endochondral ossification and endosteal mineralization through activation of the bmp-smad pathway during postnatal growth. Bone Rep (2021) 15:101088. doi: 10.1016/j.bonr.2021.101088 34141832 PMC8188257

[13] Schwarz C Wulsten D Ellinghaus A Lienau J Willie BM Duda GN. Mechanical load modulates the stimulatory effect of Bmp2 in a rat nonunion model. Tissue Eng Part A (2013) 19(1-2):247–54. doi: 10.1089/ten.TEA.2012.0265 PMC3530931 22861354

[14] Hustedt JW Blizzard DJ. The controversy surrounding bone morphogenetic proteins in the spine: A review of current research. Yale J Biol Med (2014) 87(4):549–61. PMC4257039 25506287

[15] Zerath E Holy X Noël B Malouvier A Hott M Marie PJ. Effects of bmp-2 on osteoblastic cells and on skeletal growth and bone formation in unloaded rats. Growth Horm IGF Res (1998) 8(2):141–9. doi: 10.1016/s1096-6374(98)80104-4 10987681

[16] Childress P Brinker A Gong CS Harris J Olivos DJ 3rd Rytlewski JD. Forces associated with launch into space do not impact bone fracture healing. Life Sci Space Res (Amst) (2018) 16:52–62. doi: 10.1016/j.lssr.2017.11.002 29475520 PMC5828031

[17] Scofield DC Rytlewski JD Childress P Shah K Tucker A Khan F. Development of a step-down method for altering Male C57bl/6 mouse housing density and hierarchical structure: Preparations for spaceflight studies. Life Sci Space Res (Amst) (2018) 17:44–50. doi: 10.1016/j.lssr.2018.03.002 29753413 PMC6196723

[18] Rytlewski JD Childress PJ Scofield DC Khan F Alvarez MB Tucker AT. Cohousing Male mice with and without segmental bone defects. Comp Med (2018) 68(2):131–8. PMC5897969 29663938

[19] Maupin KA Childress P Brinker A Khan F Abeysekera I Aguilar IN. Skeletal adaptations in young Male mice after 4 weeks aboard the international space station. NPJ Microgravity (2019) 5:21. doi: 10.1038/s41526-019-0081-4 31583271 PMC6760218

[20] Chu TM Warden SJ Turner CH Stewart RL. Segmental bone regeneration using a load-bearing biodegradable carrier of bone morphogenetic protein-2. Biomaterials (2007) 28(3):459–67. doi: 10.1016/j.biomaterials.2006.09.004 PMC1986795 16996588

[21] Zamarioli A Campbell ZR Maupin KA Childress PJ Ximenez JPB Adam G. Analysis of the effects of spaceflight and local administration of thrombopoietin to a femoral defect injury on distal skeletal sites. NPJ Microgravity (2021) 7(1):12. doi: 10.1038/s41526-021-00140-0 33772025 PMC7997973

[22] Zamarioli A SS Dadwal U Childress P Kacena MA. (2020). Assessment of bone healing agents for promoting bone regeneration in spaceflight, in: Orthopaedic Research Society Annual Meeting, Volume 45. Phoenix, AZ, Feb 2020.

[23] Bouxsein ML Boyd SK Christiansen BA Guldberg RE Jepsen KJ Müller R. Guidelines for assessment of bone microstructure in rodents using micro-computed tomography. J Bone Miner Res (2010) 25(7):1468–86. doi: 10.1002/jbmr.141 20533309

[24] Tazaki J Murata M Akazawa T Yamamoto M Ito K Arisue M. Bmp-2 release and dose-response studies in hydroxyapatite and beta-tricalcium phosphate. BioMed Mater Eng (2009) 19(2-3):141–6. doi: 10.3233/bme-2009-0573 19581707

[25] Visser R Arrabal PM Becerra J Rinas U Cifuentes M. The effect of an rhbmp-2 absorbable collagen sponge-targeted system on bone formation in vivo. Biomaterials (2009) 30(11):2032–7. doi: 10.1016/j.biomaterials.2008.12.046 19155065

[26] Beederman M Lamplot JD Nan G Wang J Liu X Yin L. Bmp signaling in mesenchymal stem cell differentiation and bone formation. J BioMed Sci Eng (2013) 6(8a):32–52. doi: 10.4236/jbise.2013.68A1004 26819651 PMC4725591

[27] Qin W Liu L Wang Y Wang Z Yang A Wang T. Mir-494 inhibits osteoblast differentiation by regulating bmp signaling in simulated microgravity. Endocrine (2019) 65(2):426–39. doi: 10.1007/s12020-019-01952-7 31129811

[28] Zhang C Li L Jiang Y Wang C Geng B Wang Y. Space microgravity drives transdifferentiation of human bone marrow-derived mesenchymal stem cells from osteogenesis to adipogenesis. FASEB J (2018) 32(8):4444–58. doi: 10.1096/fj.201700208RR 29533735

[29] Klosterhoff BS Vantucci CE Kaiser J Ong KG Wood LB Weiss JA. Effects of osteogenic ambulatory mechanical stimulation on early stages of bmp-2 mediated bone repair. Connect Tissue Res (2022) 63(1):16–27. doi: 10.1080/03008207.2021.1897582 33820456 PMC8490484

[30] Yuan SG Hu HL Wang XJ Yang JC Zhou RP Bai XC. Bindarit reduces bone loss in ovariectomized mice by inhibiting Ccl2 and Ccl7 expression via the nf-Kb signaling pathway. Orthop Surg (2022) 14(6):1203–16. doi: 10.1111/os.13252 PMC9163972 35470579

[31] Eyal S Derendorf H. Medications in space: In search of a pharmacologist's guide to the galaxy. Pharm Res (2019) 36(10):148. doi: 10.1007/s11095-019-2679-3 31414302

[32] Colleran PN Wilkerson MK Bloomfield SA Suva LJ Turner RT Delp MD. Alterations in skeletal perfusion with simulated microgravity: A possible mechanism for bone remodeling. J Appl Physiol (1985) (2000) 89(3):1046–54. doi: 10.1152/jappl.2000.89.3.1046 10956349

[33] Gerbaix M Gnyubkin V Farlay D Olivier C Ammann P Courbon G. One-month spaceflight compromises the bone microstructure, tissue-level mechanical properties, osteocyte survival and lacunae volume in mature mice skeletons. Sci Rep (2017) 7(1):2659. doi: 10.1038/s41598-017-03014-2 28572612 PMC5453937

[34] Sartori R Schirwis E Blaauw B Bortolanza S Zhao J Enzo E. Bmp signaling controls muscle mass. Nat Genet (2013) 45(11):1309–18. doi: 10.1038/ng.2772 24076600

[35] Sartori R Sandri M. Bmps and the muscle-bone connection. Bone (2015) 80:37–42. doi: 10.1016/j.bone.2015.05.023 26036170

[36] Scimeca M Piccirilli E Mastrangeli F Rao C Feola M Orlandi A. Bone morphogenetic proteins and myostatin pathways: Key mediator of human sarcopenia. J Transl Med (2017) 15(1):34. doi: 10.1186/s12967-017-1143-6 28202082 PMC5310081

[37] Morey-Holton ER Globus RK. Hindlimb unloading of growing rats: A model for predicting skeletal changes during space flight. Bone (1998) 22(5 Suppl):83s–8s. doi: 10.1016/s8756-3282(98)00019-2 9600759

[38] National Research Council. Guide for the care and use of laboratory animals. Washington DC: The National Academies Press (2011). Available at: https://grants.nih.gov/grants/olaw/guide-for-the-care-and-use-of-laboratory-animals.pdf.

